# Alterations in the rumen bacterial communities and metabolites of finishing bulls fed high-concentrate diets supplemented with active dry yeast and yeast culture

**DOI:** 10.3389/fmicb.2022.908244

**Published:** 2022-12-20

**Authors:** Kai Gao, Chunyin Geng

**Affiliations:** ^1^College of Agriculture, Yanbian University, Yanji, China; ^2^Engineering Research Center of North-East Cold Region Beef Cattle Science & Technology Innovation, Ministry of Education, Yanbian University, Yanji, China

**Keywords:** cattle, rumen bacteria, metabolome, active dry yeast, yeast culture

## Abstract

This study investigated the effects of active dry yeast (ADY) and yeast culture (YC) supplementation on rumen bacteria and metabolites in finishing bulls fed high-concentrate diets using the full-length 16S rDNA gene sequencing and liquid chromatography-mass spectrometry. Supplementation with ADY improved the alpha diversity and relative abundance of rumen bacteria, while YC only affected relative abundance of rumen bacteria at the genus level. Sixty-three differential metabolites were identified in rumen fluid after ADY supplementation, and 17 after YC. PICRUSt2 functional prediction showed that ADY supplementation improved the capacity of amino acid metabolism, lipid metabolism, carbohydrate metabolism, metabolism of terpenoids and polyketides, and energy metabolism in rumen bacteria (all *P* < 0.05). Correlation analysis showed that the rumen differential metabolites following ADY supplementation were mainly related to *Oligosphaera*, *Verruc*, *Mycoplasma*, and *Anaeroplasma*. Supplementation with ADY was more effective than YC in remodeling the rumen bacterial flora structure and metabolite composition under high-concentrate diets.

## 1 Introduction

Yeast preparations from *Saccharomyces cerevisiae* can be classified into two types according to their live yeast cell counts, namely active dry yeasts (ADY) and yeast cultures (YC). ADY guarantee high numbers of live yeast cells (>10^9^ colony forming units/g) and are sold as 100% ADY, while YC are sold as the complete culture medium containing a small amount of live cells. ADY and YC have been used in ruminants to favorably modify the rumen environment and improve production performance. However, the literature regarding ADY and YC does not conclusively show that supplementation benefits performance at all times (Hinman et al., 1998; Castillo et al., 2006; Bontempo et al., 2009; Finck et al., 2014; Swyers et al., 2014; Geng et al., 2016). Differing experimental conditions, including the strain of yeast, dietary composition, and animal status, may explain the variable results reported (Chaucheyras-Durand et al., 2008). Our previous study also showed that supplementation of beef cattle diets with ADY or YC had different effects on growth performance and rumen fermentation under the same trial conditions, indicating the type of yeast product may have an important impact on the outcome (Geng et al., 2016). Some researchers propose that ADY acts as a probiotic while YC acts as a prebiotic in the rumen. However, comparisons of the mechanisms of action of ADY and YC under the same experimental conditions are lacking. Elucidating these mechanisms as they impact performance will be beneficial for both scientific applications and product development.

Rumen of beef cattle contains a large number of microorganisms, bacteria being the most abundant (Shabat et al., 2016; Li and Guan, 2017). A large number of metabolites are produced when rumen bacteria act on fermentation substrates. This process can meet 70% of the energy needs of the host (Li and Guan, 2017), while the metabolites facilitate information exchange between rumen microorganisms and the host (Nicholson et al., 2012). Thus, the study of the rumen bacteria and their metabolites is a prerequisite to exploring the interaction between yeast preparations and host performance. However, there is little comparative research on the effects of ADY and YC supplementation (as two typical commercially available preparations) on rumen microflora and metabolites under common conditions in beef cattle. This study compared the effects of ADY and YC supplementation on the rumen bacterial communities and metabolites of finishing bulls fed high-concentrate diets in order to elucidate the mechanisms of typical yeast preparations on the rumen microenvironment and growth performance. Growth performance, ruminal fermentation parameters, fatty acid composition, carcass traits and blood indexes were reported previously (Geng et al., 2016, 2018a,b).

## 2 Materials and methods

The experimental design and animal management were approved by the Animal Ethics Committee of Yanbian University (Yanji, China) and carried out in accordance with the Guide for the Care and Use of Laboratory Animals (8^th^ edition, National Academies Press).

### 2.1 Animals, experimental design, and sample collection

The experimental design was as described in our previous study (Geng et al., 2016). Briefly, 45 healthy finishing cattle (Simmental × Chinese Luxi yellow bulls) aged 24 months with mean bodyweight of 505 ± 29 kg were randomly divided into three groups. The control group (MC) was fed a basal diet, the ADY group (ML) was fed basal diet supplemented with ADY preparation (Levucell SC, *S. cerevisiae* CNCM1-1077; 0.8 g/head/day; white; >0.8 × 10^10^ CFU/g), and the YC group (MY) was fed basal diet supplemented with YC preparation (Diamond V XP, Cedar Rapids, IA, USA; 50 g/head/day). The basal diet was a total mixed ration (TMR; concentrate to forage ratio 70:30) and its ingredients and nutritional composition are shown in Supplementary Table 1. The trial lasted for 98 days and bulls were fed twice a day at 05:00 and 17:00. At the end of the trial, all bulls were fasted for 24 h, and then slaughtered by captive bolt stunning and exsanguination. Eleven rumen fluid samples were randomly selected from each group (100 ml per bull, filtered through four layers of sterile gauze, and stored in 15 ml cryopreservation tubes). Rumen fluid was snap frozen in liquid nitrogen and stored at −80°C.

### 2.2 Rumen bacteria DNA extraction and sequencing

Frozen rumen fluid samples were thawed in iced water and total bacterial DNA was extracted from 200 μl using a TGuide S96 Magnetic Soil/Stool DNA Kit (Tiangen Biotech Co., Ltd, Beijing, China). DNA sequencing was as described by Klemetsen et al. (2019). Briefly, DNA purity and concentration were determined with a Synergy HTX multi-mode reader (Gene Company Limited, Hong Kong, China) and DNA integrity was assessed by 1.8% agarose gel electrophoresis. After quantitative measurement of DNA, 27 samples were qualified (nine samples in each group) and the rests were discarded due to low DNA quality. The full-length 16S rDNA sequence was amplified using the universal primers: 27F (5′-AGRGTTTGATYNTGGCTCAG-3′) and 1492R (5′-TASGGHTACCTTGTTASGACTT-3′). The PCR reaction system contained the barcode primer pair (3 μl), genomic DNA (1.5 μl), nuclease-free water (10.5 μl), and KOD OneTM PCR Master Mix (15 μl) (Karlsen et al., 2014). The cycling parameters were as follows: initial denaturation for 2 min at 95°C, then 98°C for 10 s, 55°C for 30 s, and 72°C for 90 s, for 25 cycles, and a final extension for 5 min at 72°C. PCR amplification products were detected by Qubit4 fluorometer (Thermo Fisher, New York, USA) and 1.8% agarose gel electrophoresis, before purification, quantification, and homogenization to create a sequence library. The marker genes were sequenced by single molecule real-time sequencing using a PacBio Sequel II system (Pacific Biosciences, Menlo Park, CA, United States).

### 2.3 Sequence data processing and analysis

Analysis of sequence data followed Yuan et al. (2021). Effective reads were obtained by filtering the raw reads using Trimmomatic (v.0.33), identification and removal of primer sequences by cutadapt (v.1.9.1), splicing of high-quality reads by FLASH (v.1.2.7), and removal of potential chimera using the Uchime algorithm. Operational Taxonomic Units (OTUs) were obtained by clustering reads at 97.0% similarity level using Usearch (v.10.0). Taxonomic annotation of OTUs based on the SILVA database (Release 132) used the naive Bayes classifier. Species abundance at phylum and genus levels was generated by QIIME2 (v.2020.6) and mapped by R (v.3.3.2). The alpha diversity indices (Chao1, ACE, Shannon, and Simpson) were evaluated using QIIME2 (v.2020.6). Shannon curves and species accumulation curves (OTU level) were created using Mothur software and R (v.3.3.2). QIIME2 (v.2020.6) was used to determine beta diversity, the Bray Curtis algorithm to calculate the distance between samples to obtain the beta value, and principal component analysis (PCA) and principal coordinates analysis (PCoA) for dimension reduction ranking analysis of beta diversity. Species abundance data between groups were analyzed using Metastats software and species were screened according to *P*-value or *Q*-value. Functional gene prediction analysis was based on Parks et al. (2014). Briefly, characteristic sequences were annotated using PICRUSt2 and matched with the Kyoto Encyclopedia of Genes and Genomes Database (KEGG) to predict the functional gene composition of a sample. STAMP software was used to carry out *t*-tests on functional abundances between groups.

### 2.4 Liquid chromatography-mass spectrometry (LC-MS) metabolomics

Rumen fluid (100 μl), methanol (300 μl; CAS: 67-56-1; CNW Technologies) and internal standard (20 μl) were mixed in a sterile 1.5 ml Eppendorf (EP) tube. After ultrasonication for 10 min (iced water bath), the tube was stored at −20°C for 1 h. After centrifugation (4°C, 13,000 rpm, 15 min), 200 μl of supernatant was placed in a 2 ml LC-MS autosampler vial with glass insert. Supernatant (20 μl) from each sample was also mixed in a quality control (QC) sample for monitoring instrument status, balancing the LC-MS system, and identifying metabolites. An Agilent 1290 Infinity II ultra-high performance liquid chromatography system equipped with a UPLC BEH Amide column (1.7 μm × 2.1 mm × 100 mm; Waters), and an ABSCIEX TripleTOF 5600 mass spectrometer with Analyst TF software (v.1.7; ABSCIEX) was used. Samples were eluted in water containing 25 mM ammonium acetate and 25 mM ammonia (mobile phase A) and acetonitrile (mobile phase B). A flow rate of 0.50 ml/min was applied to the elution gradient (A%:B%) 5:95 for 0–0.5 min, 35:65 at 7 min, 60:40 at 8 min, and 5:95 at 9.10 min, followed by re-equilibration for 2.9 min. The bombardment energy was 30 eV (15 secondary spectra every 50 ms). Parameters for the electrospray ion source were as follows: atomization pressure (GAS1) 60 psi, auxiliary pressure (GAS2) 60 psi, air curtain pressure 35 psi, temperature 650°C, and spray voltage 5,000 V (positive ion mode) or -4000 V (negative ion mode). Raw MS data were converted to mzXML format using ProteoWizard software. XCMS^1^ was used for retention time correction, and peak recognition, extraction, integration, and alignment. After normalizing the total peak area, metabolites were preliminarily identified in an in-house secondary mass spectrometry database using R (v.3.3.2), then compared with KEGG, Human Metabolome Database (HMDB), Lipid Maps, and a self-built database on the BioMarke Cloud Platform^2^ to identify known metabolites.

### 2.5 Metabolomics data analysis

Samples of 16S rDNA sequences were selected for statistical analysis of metabolites to ensure the reliability of the results. Metabolites were analyzed by PCA to illustrate preliminary differences between groups. Spearman rank correlation coefficient (*r*^2^) was used to evaluate the correlation of biological repetition among samples. Metabolite data were analyzed by orthogonal partial least squares discriminant analysis (OPLS-DA) using the “ropls” package in R (v.3.3.2). Volcano maps of known metabolites were drawn using the “ggplot2” package in R (v.3.3.2). The parameter threshold for differential metabolite screening was set at VIP (Variable Importance in the Projection) > 1.5, FC (Fold Change) > 1.5, and *P* < 0.05. Student’s *t*-test (unpaired) was used to determine significant differences.

### 2.6 Combined analysis

Our previous researches showed that YC supplementation could markedly reduce valerate molar percentage and increase acetate molar percentage in rumen fluid, and the ADY can sensibly improve the dietary dry matter intake (DMI) and average daily gain (ADG) of finishing bulls (Geng et al., 2016, 2018b). Spearman’s correlation matrix and *P*-value matrix were calculated for the differential rumen bacteria (genus level), differential metabolites, DMI, ADG, valerate molar percentage, and acetate molar percentage, visualizing the correlation heat maps using Python (v.2.7.5). Correlation coefficients ranging from −1 to +1 represented strong negative correlations to strong positive correlations. Correlation *P*-values less than 0.05 and 0.01 represented significant and extremely significant correlations, respectively.

## 3 Results

### 3.1 Diversity of rumen bacteria

**FIGURE 1:**
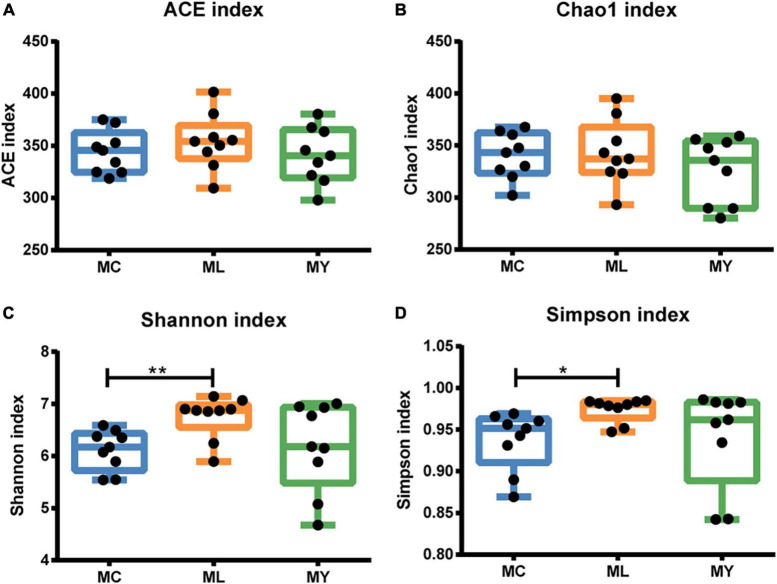
Box plots of alpha diversity indices of ruminal bacteria. **(A)** ACE, **(B)** Chao 1, **(C)** Shannon, and **(D)** Simpson index values of rumen bacteria of finishing bulls. MC, control group (*n* = 9); ML, active dry yeast group (*n* = 9); MY, yeast culture group (*n* = 9). *P < 0.05, **P < 0.01.

**FIGURE 2:**
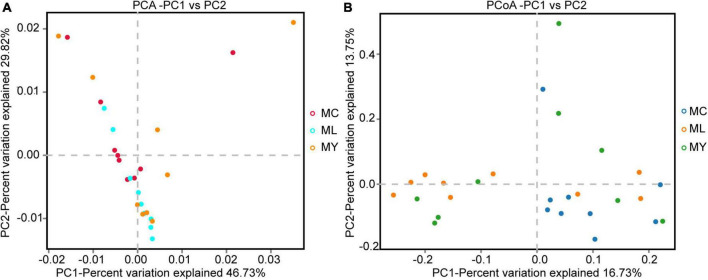
Beta diversity analysis of ruminal bacteria. **(A)** Principal component analysis (PCA), and **(B)** principal coordinates analysis (PCoA). MC, control group (*n* = 9); ML, active dry yeast group (*n* = 9); MY, yeast culture group (*n* = 9).

A total of 113,158 circular consensus sequences (CCS) were obtained by identifying barcodes in the sequencing results from 27 samples. Each sample produced at least 3061 CCS, with an average of 4191. Sequence efficiency exceeded 95% (Supplementary Figure 1). Alpha diversity analysis showed that the Shannon curve of each group had a plateau period (Supplementary Figure 2A) and the cumulative curve of species relative abundance tended to be flat (Supplementary Figure 2B), indicating that the sampling was sufficient. The Shannon (*P* < 0.01) and Simpson (*P* < 0.05) indices of ML were significantly higher than MC (Figure 1). The alpha diversity of MY was not significantly different from MC and ML (*P* > 0.05). In the PCA and PCoA plots (Figure 2), the points representing rumen bacteria in ML and MC were clustered in separate quadrants, while the MY bacteria were scattered between the ML and MC groups.

### 3.2 Composition of rumen bacteria

**FIGURE 3:**
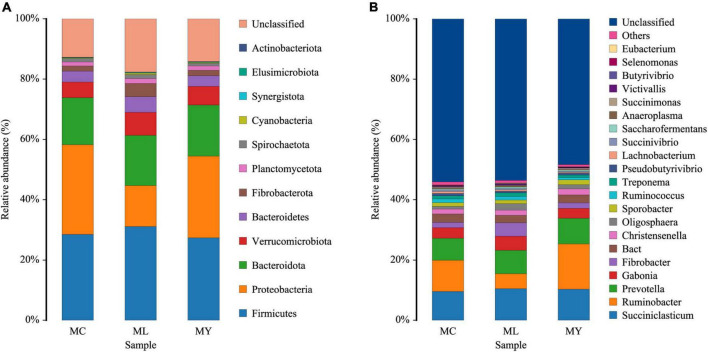
The relative abundances of ruminal bacterial community at **(A)** phylum level and **(B)** genus level (relative abundance more than 0.1%). Colors of block represent species, and the height of the block represents the proportion of the species. MC, control group (*n* = 9); ML, active dry yeast group (*n* = 9); MY, yeast culture group (*n* = 9).

**FIGURE 4:**
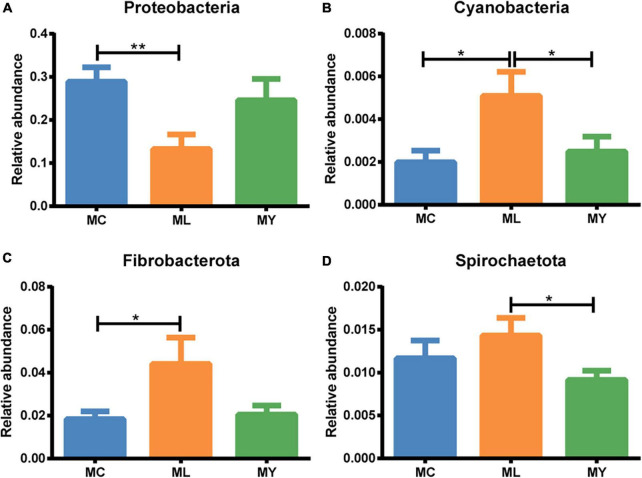
Rumen differential bacteria at phylum level (metastats analysis). The relative abundance of **(A)** *Proteobacteria*, **(B)** *Cyanobacteria*, **(C)** *Fibrobacteriota*, and **(D)** *Spirochaetota* in MC, ML, and MY groups. Data were expressed as mean ± SEM. MC, control group (*n* = 9); ML, active dry yeast group (*n* = 9); MY, yeast culture group (*n* = 9). *P < 0.05, **P < 0.01.

Twelve bacterial phyla were clustered using the Bayesian RDP classifier algorithm. Firmicutes, Proteobacteria, and Bacteroidota were the dominant phyla in the three groups (Figure 3A). Metastats analysis of bacterial abundance at phylum level (Figure 4) showed that the relative abundance of Proteobacteria in ML decreased significantly (*P* < 0.05) while Cyanobacteria and Fibrobacteriota increased significantly (*P* < 0.05) compared with MC. However, there were no significant differences at phylum level between MY and MC (*P* > 0.05).

**FIGURE 5:**
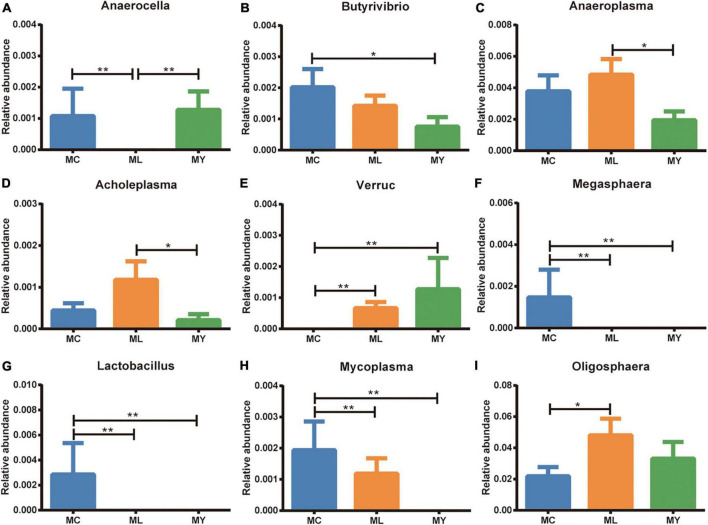
Rumen differential bacteria at genus level (metastats analysis). The relative abundance of **(A)** *Anaerocella*, **(B)** *Butyrivibrio*, **(C)** *Anaeroplasma*, **(D)** *Acholeplasma*, **(E)** *Verruc*, **(F)** *Megasphaera*, **(G)** *Lactobacillus*, **(H)** *Mycoplasma*, and **(I)** *Oligosphaera* in MC, ML. and MY groups. Data were expressed as mean ± SEM. MC, control group (*n* = 9); ML, active dry yeast group (*n* = 9); MY, yeast culture group (n = 9). *P < 0.05, **P < 0.01.

Analysis of abundance at genus level showed the dominant bacterial genera to be *Ruminobacter*, *Succiniclasticum*, *Prevotella*, *Gabonia*, and *Fibrobacter* (Figure 3B). The dominant genus was *Ruminobacter* in MC (10.33%) and MY (15.04%), and *Succiniclasticum* (10.53%) in ML. The relative abundance of *Anaerocella* in ML decreased significantly (*P* < 0.01), while *Oligosphaera* increased significantly (*P* < 0.05) compared with MC. The relative abundances of *Anaeroplasma* and *Acholeplasma* were significantly higher (*P* < 0.05) in ML than MY. Interestingly, the relative abundance of *Verruc* increased significantly in ML and MY (*P* < 0.01) compared with MC, while *Megasphaera* and *Lactobacillus* decreased significantly (*P* < 0.01) (Figure 5).

### 3.3 Functional analysis of rumen bacteria

**FIGURE 6:**
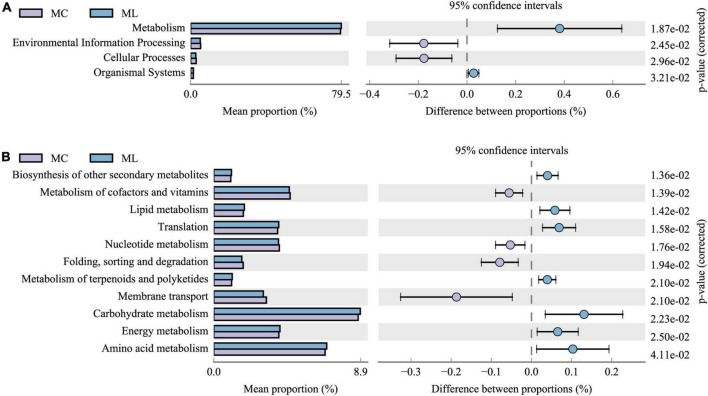
Comparison of predicted KEGG functions of rumen bacteria in ML vs MC. (Only show functions with gene abundance greater than or equal to 1%). **(A)** Functional classification level 1, and **(B)** functional classification level 2. MC, control group (*n* = 9); ML, active dry yeast group (*n* = 9).

The distribution of functional gene abundance in rumen bacteria at each functional level was predicted using PICRUSt2. Due to the low degrees of difference in gene function between MY and MC, and between MY and ML, KEGG functional analysis was carried out only between ML and MC (Figure 6). At the first functional level (Figure 6A) the metabolic ability and organic systems performance of rumen bacteria in ML were significantly improved (*P* < 0.05), while environmental information processing and cellular processes were significantly reduced (*P* < 0.05). At the second functional level (Figure 6B) the improvement of metabolic capacity was mainly reflected in the improved biosynthesis of secondary metabolites, lipid metabolism, translation, metabolism of terpenoids and polyketides, carbohydrate metabolism, energy metabolism, and amino acid metabolism (all *P* < 0.05). The decline in cellular processes was mainly reflected in the reduction of the ability of nucleus metadata, folding, sorting and degradation, and membrane transport (all *P* < 0.05).

### 3.4 Analysis of rumen metabolites

**FIGURE 7:**
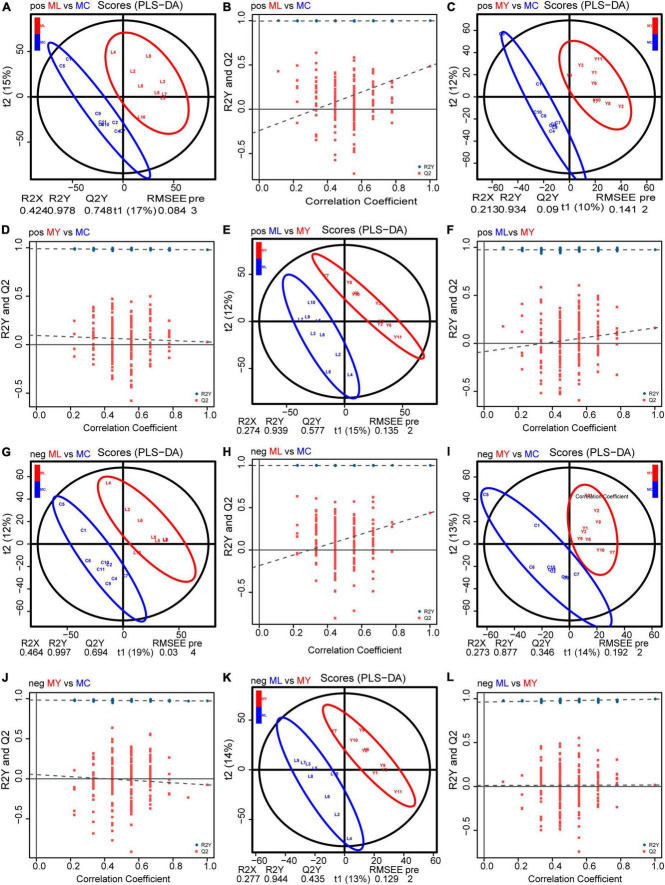
Orthogonal partial least squares discriminant analysis (OPLS–DA) plot and response permutation testing (RPT) of rumen metabolites. POS (positive ionization mode): **(A,B)** for ML vs MC; **(C,D)** for MY vs MC; **(E,F)** for ML vs MY. NEG (negative ionization mode): **(G,H)** for ML vs MC; **(I,J)** for MY vs MC; **(K,L)** for ML vs MY. R2Y and Q2 indicates the cumulative interpretation power and predictive power of the model, respectively. MC, control group (*n* = 9); ML, active dry yeast group (*n* = 9); MY, yeast culture group (*n* = 9).

**FIGURE 8:**
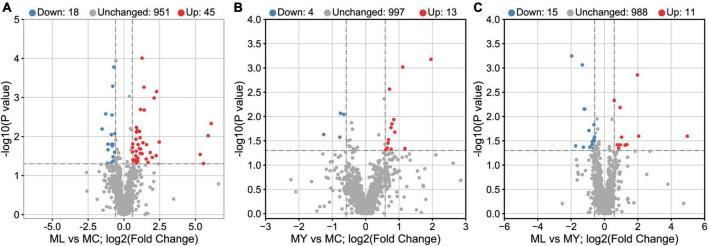
Volcano plots of rumen known metabolites in **(A)** ML vs. MC, **(B)** MY vs. MC, and **(C)** ML vs. MY. The known metabolites in positive ion mode and negative ion mode were combined. Rumen metabolites with FC (fold change) > 1.5, VIP (variable importance in the projection) > 1.5, and *P*-value < 0.05 were considered significant. Each point in the volcano plot represents a metabolite. Blue dots represent down-regulated differential metabolites, red dots represent up-regulated differential metabolites, and gray dots represent detected but insignificant metabolites. MC, control group; ML, active dry yeast group; MY, yeast culture group.

Unsupervised multivariate statistical analysis (PCA) of all samples provided an overview of rumen metabolites in the three groups (Supplementary Figure 3). Differential rumen metabolites across the groups were clearly distinguished using a supervised discriminant analysis method (OPLS-DA) (Figure 7). There were 63, 17, and 26 differential metabolites identified in ML vs. MC, MY vs. MC, and ML vs. MY, respectively (Figure 8). Of the ML vs MC differential metabolites, twelve were classified as amino acids, peptides and analogs, three as fatty acids and conjugates, two as fatty acid esters, four as eicosanoids, five as carbohydrates and carbohydrate conjugates, two as monoterpenoids, six as purine nucleosides, and eight as other lipids and lipid-like molecules (Supplementary Table 2). Of the MY vs. MC differential metabolites, three were classified as amino acids, peptides and analogs, one as fatty acid esters, one as carbohydrates and carbohydrate conjugates, one as monoterpenoids, and one as purines and purine derivatives (Supplementary Table 3). Of the ML vs. MY differential metabolites, four were classified as amino acids, peptides and analogs, two as fatty acids and conjugates, three as purines and purine derivatives, two as carbohydrates and carbohydrate conjugates, and one as other lipids and lipid-like molecules (Supplementary Table 4). The only differential metabolite common to all three groups was N-acetylhistamine. KEGG functional annotation and enrichment analysis showed that the differential metabolite pathway was rich in amino acid metabolism (e.g., lysine biosynthesis and degradation, histidine metabolism, and tryptophan metabolism), fatty acid metabolism (e.g., glycerophospholipid metabolism and arachidonic acid metabolism), glucose metabolism (e.g., glycolysis/gluconeogenesis), purine metabolism, and pyrimidine metabolism (Supplementary Figure 4).

### 3.5 Combined analysis

**FIGURE 9:**
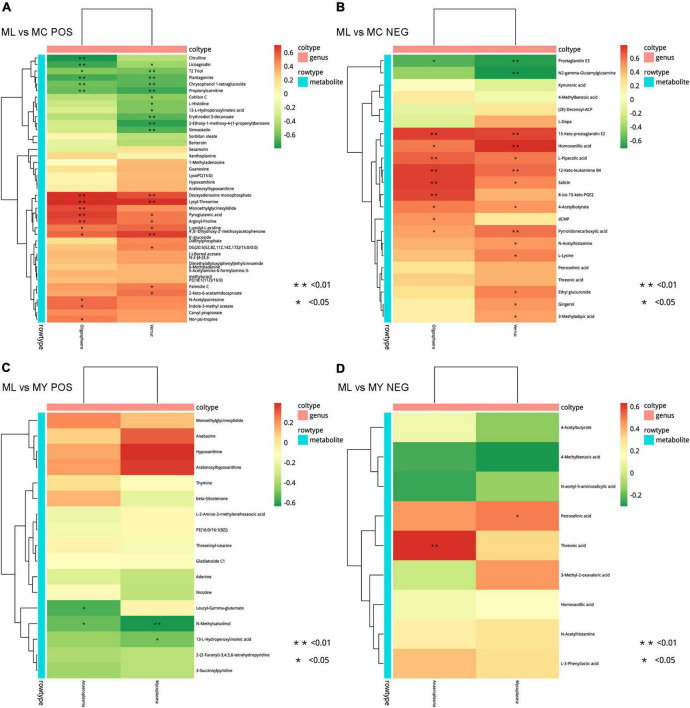
Correlation analysis between significantly differential bacteria (genus level) and differential metabolites in ML vs. MC **(A,B)** and ML vs. MY **(C,D)**. POS, positive ion mode; NEG, negative ion mode. Positive correlations are shown in red and negative correlations in green. MC, control group (*n* = 9); ML, active dry yeast group (*n* = 9); MY, yeast culture group (*n* = 9). In this analysis, rumen metabolites with FC (fold change) > 1.5, VIP (variable importance in the projection) > 1.5, and *P*-value < 0.05 were considered significant.

There were too few differential bacteria at the genus level in MY vs. MC, so correlation analysis was carried out only for ML vs. MC and ML vs. MY (Figure 9). Correlation of rumen bacteria and metabolites in ML vs. MC showed that *Oligosphaera* and *Verruc* genera were negatively associated with licoagrodin, T2 triol, plantagonine, chrysophanol 1-tetraglucoside, propionylcarnitine, and prostaglandin E3, and positively associated with deoxyadenosine monophosphate, lysyl-threonine, pyroglutamic acid, arginyl-proline, L-prolyl-L-proline, 4′,6′-dihydroxy-2′-methoxyacetophenone 6′-glucoside, 15-keto-prostaglandin E2, homovanillic acid, L-pipecolic acid, 12-keto-leukotriene B4, salicin, 4-acetylbutyrate, and pyrrolidonecarboxylic acid. The genus *Oligosphaera* was also negatively associated with citrulline and positively associated with monoethylglycinexylidide, N-acetylputrescine, indole-3-methyl acetate, nor-psi-tropine, dCMP, and 8-iso-15-keto-PGE2. The genus *Verruc* was also negatively associated with cohibin C, L-histidine, 13-L-hydroperoxylinoleic acid, erythrodiol 3-decanoate, 2-ethoxy-1-methoxy-4-(1-propenyl)benzene, simvastatin, and N2-gamma-glutamylglutamine, and positively associated with DG(20:5(5Z,8Z,11Z,14Z,17Z)/15:0/0:0), palmidin C, 2-keto-6-acetamidocaproate, N-acetylhistamine, L-lysine, ethyl glucuronide, gingerol, and 3-methyladipic acid. Correlation analysis of ML vs MY showed that *Anaeroplasma* and *Mycoplasma* were significantly negatively associated with N-methylsalsolinol. *Anaeroplasma* was negatively associated with leucyl-gamma-glutamate and positively associated with threonic acid, while *Mycoplasma* was negatively associated with 13-L-hydroperoxylinoleic acid and positively associated with petroselinic acid.

Besides, as shown in Supplementary Figure 5, the relative abundance of *Verruc* (*r* = 0.47, *r* = 0.39; *P* < 0.05) was positively correlated with ADG and acetate molar percentage, and the relative abundance of *Lactobacillus* (*r* = 0.40; *P* < 0.05), *Megasphaera* (*r* = 0.42; *P* < 0.05), and *Mycoplasma* (*r* = 0.42; *P* < 0.05) were positively correlated with valerate molar percentage in rumen.

## 4 Discussion

In this study, sequencing results showed that supplementation with ADY significantly increased rumen bacterial diversity, while YC supplementation had no such effect. Live yeast cells consume trace oxygen attached to food particles in the rumen, forming an anaerobic environment and rapidly fermenting rumen substrates. This competitive inhibition with other microorganisms may be the mechanism by which ADY supplementation affects microbial diversity (Chaucheyras-Durand et al., 2008). By comparison with ADY, YC contains only a few yeast cells, which may be one reason why YC did not affect rumen bacterial diversity. Nevertheless, no significant difference in rumen bacterial diversity index was observed between YC and ADY, indicating that the metabolites of active yeast found in YC may play a key role in regulating the rumen environment. Liu et al. (2021) found that ADY supplementation of low-concentrate diets at 4 g/head/day reduced the Shannon and Simpson indices. This inconsistent result may be related to the dose of supplementary ADY or the basal diets employed (Liu et al., 2017). Further exploration is needed of the specific regulatory mechanisms of ADY and YC on the diversity of rumen bacteria and their optimal doses under different dietary conditions.

Following supplementation with yeast (ADY or YC), Firmicutes, Proteobacteria, and Bacteroidota were the dominant bacterial phyla in beef cattle. This was consistent with previous studies (Jami and Mizrahi, 2012; Klevenhusen et al., 2017). However, ADY supplementation significantly decreased the relative abundance of Proteobacteria in the rumen and increased Fibrobacterota. YC supplementation had no effect on the relative abundance of bacterial phyla. Some of Proteobacteria are readily degraded, producing lipopolysaccharide after death. This lipopolysaccharide can destroy the mucosal barrier of the gastrointestinal tract (Plaizier et al., 2012; Shin et al., 2015). A plausible corollary is that ADY supplementation may lead to lower concentrations of bacterial endotoxins and, consequently, a healthier gastrointestinal tract. However, long-term ADY supplementation may be necessary to ensure a stable intestinal population of Proteobacteria because short-term ADY supplementation for 21 days tended to increase levels of lipopolysaccharide in rumen fluid (Uyeno et al., 2017). Fibrobacterota is considered the main bacteria that degrade dietary starch polysaccharides and wood fiber (Ransom-Jones et al., 2012), which implies that ADY supplementation may improve the catabolism, absorption, and utilization of dietary carbohydrates (starch and fiber polysaccharides). Similarly, previous studies indicate that addition of ADY can improve the abundance of fibrinolytic enzyme genes in rumen microorganisms and the relative abundance of bacteria related to rumen fiber digestion (AlZahal et al., 2017; Ogunade I. et al., 2019). Increased abundance and activity of cellulose-degrading bacteria is reported to decrease NH_3_-N concentration in the rumen (Chaucheyras-Durand and Fonty, 2001). The decrease of NH3-N concentration in rumen was also observed following ADY supplementation (Supplementary Table 5). Furthermore, PICRUSt2 functional prediction showed that the gene abundance representing the carbohydrate metabolism capacity of rumen bacteria was up-regulated by ADY supplementation. Compared with YC, ADY also increased the relative abundance of rumen *Spirochaetota*, which also degraded fiber polysaccharides (Warnecke et al., 2007). These findings demonstrated that ADY played a greater role in the regulation of rumen bacteria at the phylum level than did YC under these experimental conditions. This may explain why ADY, rather than YC, improved growth performance in bulls in our previous report (Supplementary Table 6; Geng et al., 2016).

At the genus level, ADY supplementation led to the dominant bacterial genus shifting from *Ruminobacter* to *Succiniclasticum*. *Ruminobacter* are obligate anaerobes that degrade soluble carbohydrates such as maltose, maltodextrins, and starch in the rumen (Anderson, 1995). *Succiniclasticum* are propionate-producing ruminal bacteria that participate in the synthesis and metabolism of glucose in ruminants (van Gylswyk, 1995). These changes in *Ruminobacter* and *Succiniclasticum* relative abundance indicate that ADY supplementation can alter the fermentation pattern of saccharides, which may be the mechanism by which ADY stabilizes rumen pH under high-grain diets. Meanwhile, ADY and YC supplementation both down-regulated the relative abundance of *Lactobacillus* and *Megasphaera*, while YC reduced *Butyrivibrio*. Under high-grain diets, *Lactobacillus* and *Butyrivibrio* produce lactic acid by fermenting fiber polysaccharides and starch (Khor et al., 2016; Mao et al., 2016). Excessive lactic acid reduces rumen pH—an important factor leading to rumen acidosis. *Megasphaera elsdenii* is the main lactic acid-utilizing bacteria in the rumen (Calsamiglia et al., 2012). Previous studies showed that both ADY and YC supplementation stabilize rumen pH in ruminants fed high-concentrate diets (Erasmus et al., 1992; Thrune et al., 2009). The underlying mechanism of the regulation of rumen pH by ADY and YC under high-grain diets in beef cattle was the reduction of lactic acid production rather than the enhancement of its consumption, which agreed with Halfen et al. (2021) and Lynch and Martin (2002). ADY and YC supplementation both caused numerical increases in rumen pH value, but these were not statistically significant (Supplementary Table 5). *M. elsdenii* is also the main producer of valeric acid in the rumen (Khan, 2006). Thus, the decreased concentration of valeric acid following yeast supplementation may be related to the down-regulation of *Megasphaera* (Supplementary Table 5). However, Malekkhahi et al. (2016) and Carpinelli et al. (2021) reported that ADY and YC supplementation tended to increase the relative abundance of *Megasphaera* in dairy cows. The cause of these inconsistent findings needs to be explored but may be related to the status of the experimental animals or dietary composition. Supplementation with ADY and YC also up-regulated the relative abundance of *Verruc*, while down-regulating *Mycoplasma*. *Verruc* contains many genes encoding broad-spectrum glycoside hydrolase, sulfatase, peptidase, carbohydrate lyase, and esterase (Martinez-Garcia et al., 2012). In the current study, the positive correlation between the relative abundance of *Verruc* and ADG was observed, which indicated that the increase of the abundance of *Verruc* was more conducive to the improvement of the growth performance of cattle. However, some active peptides encoded by the type III secretion system structural genes of *Verruca* are toxic to eukaryotic yeast (Sait et al., 2011), *Drosophila melanogaster*, and *Caenorhabditis elegans* (McGroty et al., 2013). *Mycoplasma* are considered normal flora, but some species are found in both healthy and diseased individuals (Gilroy and Taylor-Robinson, 1998). *Mycoplasma* may have an ecological association with cellulolytic anaerobic fungi in the rumen (Kudo et al., 1990). These findings suggest that ADY and YC supplementation may directly or indirectly regulate rumen fungi and protozoa. ADY supplementation also down-regulated the relative abundance of *Anaerocella*, while up-regulating *Oligosphaera*. *Anaerocella* and *Oligosphaera* are anaerobic bacteria that ferment carbohydrates to small amounts of short-chain fatty acids *in vitro* (e.g., acetate and propionate) (Abe et al., 2012; Qiu et al., 2013). Compared with YC, ADY supplementation up-regulated the relative abundance of *Anaeroplasma* and *Acholeplasma*. The relative abundance of *Anaeroplasma* may be related to the increased consumption of resistant starch that is not easily digested by cattle (Lau et al., 2018). *Acholeplasmas* are ubiquitous saprophytic bacteria in plants and animals (Siewert et al., 2014). The understanding of the roles of *Anaeroplasma* and *Acholeplasma* in the rumen is limited and further study is required.

The metabolomic data indicated that both ADY and YC supplementation up-regulated the concentration of some dipeptides, including L-prolyl-L-proline, arginyl-proline, lysyl-threonine, leucyl-gamma-glutamate, and leucyl-valine. Peptides are intermediates in ruminal protein degradation and are digested further and absorbed by the small intestine for the subsequent synthesis of protein and regulation of related metabolic pathways (Mariz et al., 2018). Dipeptides have been shown to be more effective than isoacids and amino acids for improving neutral detergent fiber digestion (Yang, 2002), which may explain the improved protein and fiber metabolism following ADY and YC supplementation (Kumar et al., 1994, 1997). Higher concentrations of N-acetylhistamine were also observed after supplementing with ADY and YC. Histidine is known to decarboxylate to form histamine under the action of histidine decarboxylase, then forms N-acetylhistamine under the action of transferases (Dempsey et al., 2015). Excessive histamine can cause pathological phenomena such as diarrhea, asthma, hypotension, arrhythmia, and skin pruritus in animals (Baba et al., 1998; Wöhrl et al., 2004), but N-acetylhistamine has no such toxicity, even in large doses (Field et al., 1991). Thus, the increased N-acetylhistamine concentrations implies that both ADY and YC supplementation enhance histidine metabolism (ko00340)—which is confirmed by KEGG functional annotation and enrichment analysis—and that supplementation will have a favorable impact on animal health. However, decreased L-histidine concentration was only observed after ADY supplementation, not YC. Correlation analysis of bacteria and metabolites showed that the N-acetylhistamine was positively correlated with the genus *Verruc* (Figure 9). Given the complex relationship between rumen bacteria and their metabolites, further verification is needed.

Active dry yeast supplementation affected the concentration of individual amino acids and their metabolites, increasing concentrations of L-lysine, 2,6-diaminopimelate, L-pipecolic acid, and pyroglutamic acid, and decreasing citrulline and N2-gamma-glutamyl glutamine. Lysine in rumen fluid is derived mainly from dietary degradation and synthesis by rumen microorganisms (Kinzel and Bhattacharjee, 1979). Increased lysine concentration means more will enter the small intestine, which is beneficial to the growth of beef cattle. 2,6-Diaminopimelate is synthesized mainly by bacteria and is incorporated into peptidoglycan in bacterial cell walls (Ghuysen, 1968). It is also used for lysine synthesis by rumen bacteria and protozoa (Onodera, 1986), which may explain the increased lysine concentration after ADY supplementation. Pipecolic acid is a major intermediate of lysine metabolism and has been isolated from the incubation medium of rumen ciliate protozoa (Onodera and Kandatsu, 1972). It can be absorbed by the rumen and/or intestine and has hypotonic and sedative effects through increasing the release of 7-aminobutyric acid in the central nervous system (Gutiérrez and Delgado-Coello, 1989). This, therefore, may constitute direct evidence that ADY supplementation can alleviate stress in ruminants under extreme environmental conditions (Ma et al., 2021).

Ruminal glutamine can be convert to pyroglutamate by *Streptococcus bovis*, and gram-negative and monensin-resistant species (Russell and Chen, 1989). Short-rod, monensin-sensitive bacteria then convert pyroglutamate to acetate and butyrate (Chen and Russell, 1989). In the present study, glutamine concentrations did not increase after ADY supplementation, indicating that the pyroglutamine biosynthesis pathway was enhanced in rumen microorganisms. Correlation analysis showed this may be related to increased relative abundance of *Oligophaera* and *Verruc*. Pyroglutamate is a component of hypothalamic-releasing hormones such as thyrotropin-releasing hormone (Russell et al., 1991). ADY supplementation has been shown to significantly increase the concentration of circulating ghrelin (a hypothalamic-releasing hormone) in bulls (Geng et al., 2018a). These results indicate that ADY supplementation impacts glutamine metabolism in rumen bacteria by affecting the secretion of hypothalamic-releasing hormones. Unlike L-glutamine, citrulline is not metabolized in the rumen but is absorbed by the small intestine for the synthesis of arginine (Wu et al., 2018). Citrulline is a transient intermediate product of arginine metabolism in rumen bacteria and ciliate protozoa (Van Den Hende et al., 1964; Onodera et al., 1983). Furthermore, the presence of protozoa can inhibit arginine metabolism in rumen microorganisms by reducing the production of 5-aminovaleric acid and ornithine from proline and citrulline (Sultana et al., 2003). The addition of ADY can reduce the protozoa population in beef cattle (Phesatcha et al., 2021). These results indicate that ADY’s effect on citrulline may be related to its effect on the abundance and activity of rumen protozoa.

This study also found that ADY supplementation increased concentrations of monoethylglycinexylidide, (2E)-decenoyl-ACP, and L-DOPA. Monoethylglycinexylidide is an active metabolite of lidocaine and is only found in individuals that have used this local anesthetic (Ben-Zvi et al., 1995). Interestingly, lidocaine was not fed to or injected into the cattle in this study and, to date, there are no reports of rumen microorganisms producing lidocaine, so follow-up research of this finding is warranted. (2E)-Decenoyl-ACP (cyclo-leucine) and L-DOPA are non-proteinogenic amino acids. Cyclo-leucine is positively correlated with meat flavor (Grabež et al., 2019), but our previous study showed that ADY supplementation did not change the flavor of beef (Geng et al., 2018b). The cyclo-leucine concentration may not have reached the threshold for affecting flavor. L-DOPA is found naturally in both animals and plants (Hornykiewicz, 2002; Soares et al., 2014), and is synthesized from L-tyrosine by the enzyme tyrosine hydroxylase. It is a precursor of some catecholamines (e.g., noradrenaline, adrenaline, and dopamine) and can increase the secretion of growth hormone in steers (Kasuya et al., 2017), which may explain why ADY supplementation helps to improve feed intake and growth performance of beef cattle (Geng et al., 2016).

The concentrations of petroselinic acid, 3-methyladipic acid, and 4-acetylbutyrate increased, and propionylcarnitine and sorbitan oleate decreased after ADY supplementation. Petroselinic acid is a long-chain unsaturated fatty acid that occurs naturally in several vegetable oils (Matloup et al., 2017), and inhibits the production of toxic substances and reduces arachidonic acid concentration in tissue lipids (Weber et al., 1995; Ramanathan et al., 2018). 3-Methyladipic acid is produced by rumen microorganisms catabolizing phytic acid, while 4-acetylbutyrate (butyrate with an aliphatic tail) inhibits apoptosis and promotes cell growth (Harfoot, 1981; Ooi et al., 2010). Propionylcarnitine is an acylcarnitine that transports acyl-groups (organic acids and fatty acids) from the cytoplasm into the mitochondria, producing energy to enhance fatty acid and branched-chain amino acid metabolism (Violante et al., 2013). Sorbitan oleate is the product of sorbitan and oleic acid esterification and acts as a surfactant and emulsifier (Xu et al., 2003). These changes in lipid metabolites demonstrate that ADY supplementation affects ruminal lipid metabolism (Amin and Mao, 2021). Moreover, the concentrations of prostaglandin (8-iso-15-keto-PGE2; log2FC = 0.74), 15-keto-prostaglandin E2 (log2FC = 0.93), prostaglandin E3 (log2FC = −0.64), and leukotrienes (12-keto-leukotriene B4; log2FC = 1.56) are affected by ADY supplementation. These are bioactive lipid mediators that play important roles in reproduction and immunity in ruminants (Trevisi et al., 2011). However, only the concentration of hexadecanedioic acid mono-L-carnitine ester (log2FC = 0.79) changed after YC supplementation. This compound has a beneficial effect in alleviating biological aging (Hagen et al., 1998). This partly explains how ADY and YC supplementation improved plasma lipid metabolism in beef cattle in our previous study (Geng et al., 2016) and how regulation of rumen fatty acids metabolism is greater with ADY supplementation than with YC under the same conditions.

Changes to carbohydrate and carbohydrate conjugate concentrations in the rumen were observed in this study. The levels of ethyl glucuronide, threonic acid, salicin, and 4′,6′-dihydroxy-2′-methoxyacetophenone 6′-glucosidein increased, while chrysophanol 1-tetraglucoside decreased after ADY supplementation. It is worth noting that these are all glycosides that can be decomposed by fermentation or enzymes to produce glucose that provides metabolic energy for both the host animal and rumen microorganisms, and they possess various bioactivities in the regulation of host physiological functions (Han et al., 2011). For instance, threonic acid participates in the ascorbic acid and aldonic acid metabolic pathway (ko00053) by acting as the reaction substrate of L-threonate 3-dehydrogenase, improving the animal’s antioxidant status. *Anaeroplasma* showed a significant positive correlation with threonic acid in this study, which implies that *Anaeroplasma* species may be key to metabolizing threonic acid. Furthermore, microcrystalline cellulose concentration decreased after YC supplementation, which has positive effects on gastrointestinal physiology and expression of lipid metabolism enzymes (Nsor-Atindana et al., 2017). This demonstration of ADY and YC supplementation affecting rumen carbohydrate metabolism is in agreement with Enjalbert et al. (1999) and Ogunade et al. (2019).

Active dry yeast and YC supplementation both increased the concentration of 6-methyladenine, while ADY increased concentrations of guanosine, hypoxanthine, 1-methyladenosine, arabinosylhypoxanthine, and deoxyadenosine monophosphate. These metabolites are closely related to rumen nitrogen metabolism (Fujihara and Shem, 2011). Xanthine and hypoxanthine are regarded as biomarkers of microbial protein synthesis (Ametaj et al., 2010). In the present study, only ADY increased hypoxanthine concentration in rumen fluid, implying that ADY is more conducive to microbial protein synthesis.

The concentration of some flavonoids also increased significantly after supplementation with ADY (e.g., licoagrodin) or YC (e.g., silandrin). Flavonoids have antioxidant, anti-inflammatory, antimutation, and anticarcinogenic properties and the ability to regulate the functions of key cellular enzymes, which improves rumen antioxidant and metabolic capacity in beef cattle (Olagaray and Bradford, 2019). Moreover, some antiviral and anti-inflammatory substances were also significantly increased by ADY and YC supplementation, for example, 2-keto-6-acetamidocaproate, lysoPC (15:0), and (-)-bornyl acetate. Those findings further demonstrate that ADY and YC supplementation affect the physiological state of beef cattle by regulating metabolites (Chaucheyras-Durand et al., 2008; Amin and Mao, 2021).

Accumulating evidence indicates that the interaction of rumen microorganisms and rumen metabolites can shape phenotypic traits in the host (Mao et al., 2016; Hua et al., 2017; Xue et al., 2020). Due to the small fluctuations in rumen bacteria following YC supplementation, correlation analysis between differential rumen bacteria and metabolites was carried out only for ML vs. MC and ML vs. MY. At the genus level, ADY affected the production or utilization of particular metabolites by regulating the relative abundance of *Oligosphaera*, *Verruc*, *Anaeroplasma*, and *Mycoplasma*. Comprehensive analysis of the rumen microbiome and metabolome demonstrated that ADY supplementation regulated the nutrition and health of finishing bulls by intervening in the rumen microbiome, but YC supplementation did not.

## 5 Conclusion

Although changes in rumen bacterial microbiota and metabolites were observed in response to both ADY and YC supplementation, ADY was more effective than YC in remodeling the bacterial flora structure and metabolite composition under high-concentrate diets. These findings provide a foundation for the rational use of ADY and YC in ruminant diets. Further research is needed into the effects of ADY and YC supplementation on rumen fungi and protozoa in beef cattle.

## Data availability statement

The datasets that support the findings of this study are available from the corresponding author upon reasonable request. The raw data of 16S rDNA sequencing reported in this manuscript are deposited in the NCBI database (accession number PRJNA907261). Available online at: http://www.ncbi.nlm.nih.gov/bioproject/907261.

## Ethics statement

The animal study was reviewed and approved by the Animal Ethics Committee of Yanbian University (Yanji, China).

## Author contributions

KG performed the experiment, analyzed the data, and wrote the manuscript. CG conceived the study and edits for the manuscript. Both authors designed the study, contributed to the article, and approved the submitted version.

## Footnotes

1
https://xcmsonline.scripps.edu/

2
http://www.biocloud.net/
