# Supplementary material for: Alterations in the rumen bacterial communities and metabolites of finishing bulls fed high-concentrate diets supplemented with active dry yeast and yeast culture
Source: Front Microbiol. 2022 Dec 20;13:908244. doi: 10.3389/fmicb.2022.908244 (PMC9810264; doi:10.3389/fmicb.2022.908244)
Supplement: Supplementary file 1 [file Data_Sheet_1.docx]

| **Table S1.** Ingredient and nutritional composition of basal diets (% of dry matter) | | | |
| --- | --- | --- | --- |
| Ingredient composition | Content (% of DM) | Nutritional composition^1^ | Content (% of DM) |
| Corn silage | 30.61 | Dry matter, DM | 71.45 |
| Corn meal | 51.14 | Crude protein | 12.23 |
| Cottonseed meal | 7.79 | Ether extract | 2.98 |
| Soybean meal | 5.79 | Neutral detergent fibers | 25.86 |
| Salt | 0.18 | Acid detergent fibers | 15.57 |
| Sodium bicarbonate | 1.04 | Calcium | 0.63 |
| Compound premix^2^ | 3.45 | Phosphorus | 0.37 |
| Total (%) | 100.00 | Sodium chloride | 0.40 |
|  |  | NEg^3^ (Mcal/kg DM) | 1.28 |
| ^1^ The value reported for nutritional composition of diets was calculated based on the nutrient analysis from ingredient samples.  ^2^ Supplied per kilogram of product. Ca: 160 g; P: 30 g; Cu: 450mg; Zn: 1600mg; Mn: 800 mg; I: 10mg; Co: 10mg; Se: 5 mg; vitamin A: 120 000 IU; vitamin D:55000 IU; vitamin E: 400 mg; vitamin B3: 600 mg; vitamin B5: 200 mg; monesin: 1000 mg; salt: 0.065 kg.  ^3^ NEg (net energy for growth) was estimated from CNCPS (6.0) values. | | | |

| **Table S2.** Significant differential metabolites in rumen fluid of ML_vs_MC (n = 9) | | | | | |
| --- | --- | --- | --- | --- | --- |
| Name of Differential Metabolites | log2FC | *P*-value | VIP | Regulated | Mode |
| **Amino acids, peptides, and analogues** | | | | | |
| L-prolyl-L-proline | 5.34 | 0.029 | 1.96 | up | POS |
| Arginyl-Proline | 0.77 | 0.040 | 1.81 | up | POS |
| Lysyl-Threonine | 5.89 | 0.010 | 2.22 | up | POS |
| Monoethylglycinexylidide | 1.15 | 0.027 | 1.93 | up | POS |
| Pyroglutamic acid | 2.25 | 0.031 | 1.88 | up | POS |
| L-Pipecolic acid | 0.81 | 0.050 | 1.70 | up | NEG |
| (2E)-Decenoyl-ACP | 1.70 | 0.046 | 1.67 | up | NEG |
| L-Lysine | 0.63 | 0.039 | 1.68 | up | NEG |
| L-Dopa | 0.84 | 0.012 | 2.14 | up | NEG |
| L-Histidine | -0.80 | 0.017 | 2.12 | down | POS |
| Citrulline | -0.88 | 0.047 | 1.77 | down | POS |
| N2-gamma-Glutamylglutamine | -0.71 | 0.000 | 2.75 | down | NEG |
| **Fatty acids and conjugates** | | | | | |
| Petroselinic acid | 1.98 | 0.034 | 1.74 | up | NEG |
| 4-Acetylbutyrate | 1.08 | 0.020 | 1.90 | up | NEG |
| 3-Methyladipic acid | 0.88 | 0.006 | 2.27 | up | NEG |
| **Fatty acid esters** | | | | | |
| Propionylcarnitine | -0.78 | 0.001 | 2.62 | down | POS |
| Sorbitan oleate | -1.11 | 0.016 | 2.04 | down | POS |
| **Eicosanoids** | | | | | |
| 12-Keto-leukotriene B4 | 1.56 | 0.038 | 1.82 | up | NEG |
| 8-iso-15-keto-PGE2 | 0.74 | 0.046 | 1.74 | up | NEG |
| 15-Keto-prostaglandin E2 | 0.93 | 0.007 | 2.27 | up | NEG |
| Prostaglandin E3 | -0.64 | 0.008 | 2.06 | down | NEG |
| **Carbohydrates and carbohydrate conjugates** | | | | | |
| Ethyl glucuronide | 0.62 | 0.016 | 1.99 | up | NEG |
| Threonic acid | 1.10 | 0.007 | 2.15 | up | NEG |
| Salicin | 0.68 | 0.049 | 1.68 | up | NEG |
| Chrysophanol 1-tetraglucoside | -0.63 | 0.041 | 1.72 | down | POS |
| 4',6'-Dihydroxy-2'-methoxyacetophenone 6'-glucoside | 6.11 | 0.005 | 2.45 | up | POS |
| **Monoterpenoids** | | | | | |
| (-)-Bornyl acetate | 0.99 | 0.016 | 2.15 | up | POS |
| Carvyl propionate | 0.59 | 0.025 | 1.99 | up | POS |
| **Purine nucleosides** | | | | | |
| 1-Methyladenosine | 1.41 | 0.001 | 2.88 | up | POS |
| Arabinosylhypoxanthine | 2.29 | 0.001 | 2.77 | up | POS |
| Guanosine | 0.60 | 0.024 | 2.07 | up | POS |
| Deoxyadenosine monophosphate | 1.84 | 0.026 | 1.98 | up | POS |
| 6-Methyladenine | 1.34 | 0.014 | 2.22 | up | POS |
| Hypoxanthine | 2.12 | 0.001 | 2.74 | up | POS |
| **Other lipids and lipid-like molecules** | | | | | |
| Cohibin C | -0.65 | 0.025 | 1.96 | down | POS |
| 13-L-Hydroperoxylinoleic acid | -1.27 | 0.003 | 2.47 | down | POS |
| DG(20:5(5Z,8Z,11Z,14Z,17Z)/15:0/0:0) | 0.60 | 0.019 | 2.00 | up | POS |
| LysoPC(15:0) | 1.17 | 0.021 | 1.91 | up | POS |
| PE(18:1(11Z)/16:0) | 1.58 | 0.016 | 2.00 | up | POS |
| Simvastatin | -1.52 | 0.006 | 2.29 | down | POS |
| T2 Triol | -0.89 | 0.009 | 2.18 | down | POS |
| Erythrodiol 3-decanoate | -0.76 | 0.033 | 1.80 | down | POS |
| **Others** | | | | | |
| Pyrrolidonecarboxylic acid | 0.84 | 0.044 | 1.74 | up | NEG |
| Palmidin C | 0.97 | 0.043 | 1.72 | up | POS |
| Xanthoplanine | 0.98 | 0.036 | 1.90 | up | POS |
| 4-Methylbenzoic acid | -1.16 | 0.048 | 1.77 | down | NEG |
| Sesamolin | 0.87 | 0.024 | 1.95 | up | POS |
| N-Acetylputrescine | 1.27 | 0.028 | 1.91 | up | POS |
| N-Acetylhistamine | 1.42 | 0.002 | 2.52 | up | NEG |
| 5-Acetylamino-6-formylamino-3-methyluracil | 0.83 | 0.031 | 1.83 | up | POS |
| Licoagrodin | -0.85 | 0.016 | 2.14 | down | POS |
| Indole-3-methyl acetate | 0.79 | 0.048 | 1.80 | up | POS |
| Berteroin | -1.11 | 0.022 | 2.02 | down | POS |
| 2-Keto-6-acetamidocaproate | 0.87 | 0.008 | 2.29 | up | POS |
| Diethylphosphate | 5.57 | 0.049 | 1.82 | up | POS |
| Homovanillic acid | 1.27 | 0.000 | 2.74 | up | NEG |
| 2-Ethoxy-1-methoxy-4-(1-propenyl)benzene | -0.83 | 0.003 | 2.48 | down | POS |
| N-2-[4-(3,3-Dimethylallyloxy)phenyl]ethylcinnamide | 1.16 | 0.002 | 2.63 | up | POS |
| Gingerol | 2.47 | 0.014 | 2.13 | up | NEG |
| Plantagonine | -0.76 | 0.042 | 1.65 | down | POS |
| dCMP | 0.73 | 0.046 | 1.70 | up | NEG |
| Kynurenic acid | -0.76 | 0.016 | 1.93 | down | NEG |
| Nor-psi-tropine | 1.13 | 0.011 | 2.23 | up | POS |
| log2FC, log2FoldChange; VIP, Variable Importance in Projection.MC, control group (n = 9); ML, active dry yeast group (n = 9). Rumen metabolites with FC (fold change) > 1.5, VIP (variable importance in the projection) > 1.5, and *P*-value < 0.05 were considered significant. POS, Positive Ion Mode; NEG, Negative Ion Mode. | | | | | |

| **Table S3.** Significant differential metabolites in rumen fluid of MY_vs_MC (n = 9) | | | | | |
| --- | --- | --- | --- | --- | --- |
| Name of Differential Metabolites | log2FC | *P*-value | VIP | Regulated | Mode |
| **Amino acids, peptides, and analogues** | | | | | |
| Leucyl-Valine | 0.84 | 0.012 | 2.54 | up | POS |
| Arginyl-Proline | 0.76 | 0.017 | 2.27 | up | POS |
| Leucyl-Gamma-glutamate | 0.69 | 0.030 | 2.17 | up | POS |
| **Fatty acid esters** | | | | | |
| Hexadecanedioic acid mono-L-carnitine ester | 0.79 | 0.014 | 2.45 | up | POS |
| **Carbohydrates and carbohydrate conjugates** | | | | | |
| Cellulose, microcrystalline | -0.65 | 0.009 | 2.58 | down | POS |
| **Monoterpenoids** | | | | | |
| Kahweol | -0.78 | 0.027 | 2.19 | down | POS |
| **Purines and purine derivatives** | | | | | |
| 6-Methyladenine | 0.67 | 0.035 | 1.94 | up | POS |
| **Others** | | | | | |
| Silandrin | 0.87 | 0.021 | 2.35 | up | POS |
| Nor-psi-tropine | 0.76 | 0.047 | 2.07 | up | POS |
| beta-Sitostenone | 1.18 | 0.046 | 2.18 | up | POS |
| Hexylamine | 0.60 | 0.048 | 1.90 | up | POS |
| N-Acetylhistamine | 0.65 | 0.045 | 1.88 | up | NEG |
| Simvastatin | -1.26 | 0.023 | 2.42 | down | POS |
| 3-Succinoylpyridine | 1.11 | 0.001 | 3.03 | up | POS |
| 2-(2-Furanyl)-3,4,5,6-tetrahydropyridine | 1.96 | 0.001 | 3.13 | up | POS |
| 2-Keto-6-acetamidocaproate | 0.72 | 0.003 | 3.04 | up | POS |
| Kynurenic acid | -0.75 | 0.009 | 2.55 | down | NEG |
| Log2FC, log2FoldChange; VIP, Variable Importance in Projection.MC, control group (n = 9); MY, yeast culture group (n = 9). Rumen metabolites with FC (fold change) > 1.5, VIP (variable importance in the projection) > 1.5, and *P*-value < 0.05 were considered significant. POS, Positive Ion Mode; NEG, Negative Ion Mode. | | | | | |

| **Table S4.** Significant differential metabolites in rumen fluid of ML_vs_MY (n = 9) | | | | | |
| --- | --- | --- | --- | --- | --- |
| Name of Differential Metabolites | log2FC | *P*-value | VIP | Regulated | Mode |
| **Amino acids, peptides, and analogues** | | | | | |
| Monoethylglycinexylidide | 1.02 | 0.027 | 2.11 | up | POS |
| Threoninyl-Leucine | -0.73 | 0.033 | 2.21 | down | POS |
| L-2-Amino-3-methylenehexanoic acid | -0.67 | 0.045 | 1.99 | down | POS |
| Leucyl-Gamma-glutamate | -0.63 | 0.029 | 1.91 | down | POS |
| **Fatty acids and conjugates** | | | | | |
| Petroselinic acid | 2.04 | 0.025 | 2.31 | up | NEG |
| 4-Acetylbutyrate | 0.85 | 0.045 | 2.18 | up | NEG |
| **Purines and purine derivatives** | | | | | |
| Hypoxanthine | 1.24 | 0.039 | 2.18 | up | POS |
| Adenine | -0.93 | 0.019 | 2.40 | down | POS |
| Arabinosylhypoxanthine | 1.34 | 0.038 | 2.20 | up | POS |
| **Carbohydrates and carbohydrate conjugates** | | | | | |
| Threonic acid | 1.98 | 0.001 | 3.25 | up | NEG |
| N-Acetylhistamine | 0.77 | 0.038 | 2.23 | up | NEG |
| **Other lipids and lipid-like molecules** | | | | | |
| 13-L-Hydroperoxylinoleic acid | -1.22 | 0.007 | 2.40 | down | POS |
| **Others** | | | | | |
| Anabasine | 4.96 | 0.025 | 2.33 | up | POS |
| 4-Methylbenzoic acid | -0.75 | 0.038 | 2.27 | down | NEG |
| N-acetyl-5-aminosalicylic acid | -1.18 | 0.007 | 2.76 | down | NEG |
| Thymine | -0.59 | 0.026 | 2.13 | down | POS |
| Gladiatoside C1 | -0.60 | 0.032 | 2.15 | down | POS |
| PE(16:0/16:1(9Z)) | -0.88 | 0.043 | 1.90 | down | POS |
| beta-Sitostenone | -1.27 | 0.043 | 2.08 | down | POS |
| 3-Succinoylpyridine | -1.33 | 0.001 | 3.08 | down | POS |
| 3-Methyl-2-oxovaleric acid | 0.95 | 0.038 | 2.32 | up | NEG |
| Homovanillic acid | 0.59 | 0.005 | 2.69 | up | NEG |
| L-3-Phenyllactic acid | 0.93 | 0.007 | 2.77 | up | NEG |
| 2-(2-Furanyl)-3,4,5,6-tetrahydropyridine | -1.97 | 0.001 | 3.41 | down | POS |
| Nicotine | -1.73 | 0.040 | 2.17 | down | POS |
| N-Methylsalsolinol | -0.63 | 0.015 | 2.37 | down | POS |
| Log2FC, log2FoldChange; VIP, Variable Importance in Projection.ML, active dry yeast group (n = 9); MY, yeast culture group (n = 9). Rumen metabolites with FC (fold change) > 1.5, VIP (variable importance in the projection) > 1.5, and *P*-value < 0.05 were considered significant. POS, Positive Ion Mode; NEG, Negative Ion Mode. | | | | | |

| **Table S5** Effect of dietary supplementation of active dry yeast and yeast culture on rumen fermentation parameters (n = 15) ^[[1]](#footnote-1)^ | | | | | |
| --- | --- | --- | --- | --- | --- |
| Items | Treatments | | | SEM | *P*-value |
|  | MC | ML | MY |  |  |
| pH | 5.68 | 5.90 | 5.88 | 0.884 | 0.17 |
| Ammonia-N (mg/100 mL) | 27.07 | 25.20 | 24.57 | 1.23 | 0.29 |
| Total VFA (mmol/L) | 123.68 | 112.19 | 108.50 | 5.46 | 0.29 |
| Acetate (%) | 61.48b | 61.82b | 65.31a | 0.94 | 0.054 |
| Propionate (%) | 22.37 | 21.67 | 19.68 | 0.97 | 0.97 |
| Isobutyrate (%) | 0.93 | 0.96 | 0.94 | 0.054 | 0.77 |
| Butyrate (%) | 11.83 | 12.30 | 11.08 | 0.51 | 0.21 |
| Isovalerate (%) | 2.29 | 2.29 | 2.10 | 0.083 | 0.25 |
| Valerate (%) | 1.12a | 1.10a | 0.89b | 0.045 | 0.012 |
| Acetate : propionate | 2.86b | 3.03ab | 3.39a | 0.13 | 0.096 |
| MC = control group; ML = active dry yeast group; MY = yeast culture group; NH_3_-N = ammoniacal nitrogen; VFA = volatile fatty acids.  ^a, b, c^ Within a row, different letters mean differed significantly (*P* < 0.05). | | | | | |

| **Table S6** Effect of dietary supplementation of active dry yeast and yeast culture on growth performance of beef cattle (n = 15) ^[[2]](#footnote-2)^ | | | | | |
| --- | --- | --- | --- | --- | --- |
| Items | Treatments | | | SEM | *P*-value |
|  | MC | ML | MY |  |  |
| Initial body weight, kg | 514 | 516 | 513 | 4.26 | 0.977 |
| Final body weight, kg | 577b | 611a | 587ab | 9.57 | 0.044 |
| Dietary dry matter intake, kg/day | 0.64b | 0.99a | 0.74ab | 0.097 | 0.044 |
| Average daily gain, kg/day | 8.7b | 10.4a | 9.4ab | 0.25 | 0.015 |
| Feed conversion (feed: gain) | 13.5 | 10.6 | 12.7 | 0.59 | 0.146 |
| MC = control group; ML = active dry yeast group; MY = yeast culture group.  ^a, b, c^ Within a row, different letters mean differed significantly (*P* < 0.05). | | | | | |


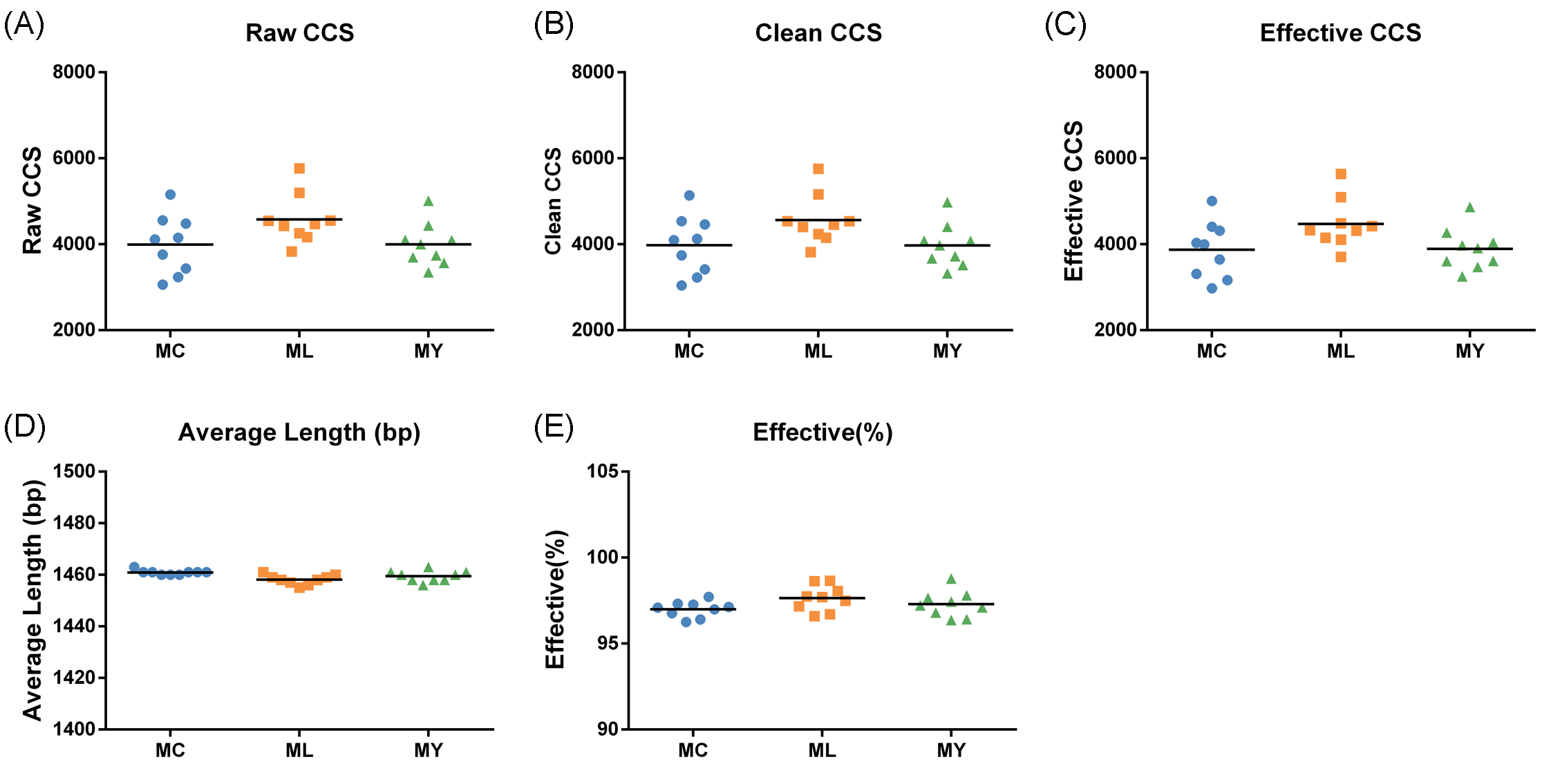


**Figure S1.** Summary of sequence statistics for all samples. (A) Raw reads represent the number of original reads. (B) Clean reads are the number of high-quality reads obtained after quality control and splicing. (C) Effective reads indicate the number of effective sequences with non-chimeras. (D) Average Length (bp) is the average sequence length of all samples. (E) Effective (%) is the percentage of effective reads in raw reads. MC, control group (n = 9); ML, active dry yeast group (n = 9); MY, yeast culture group (n = 9).


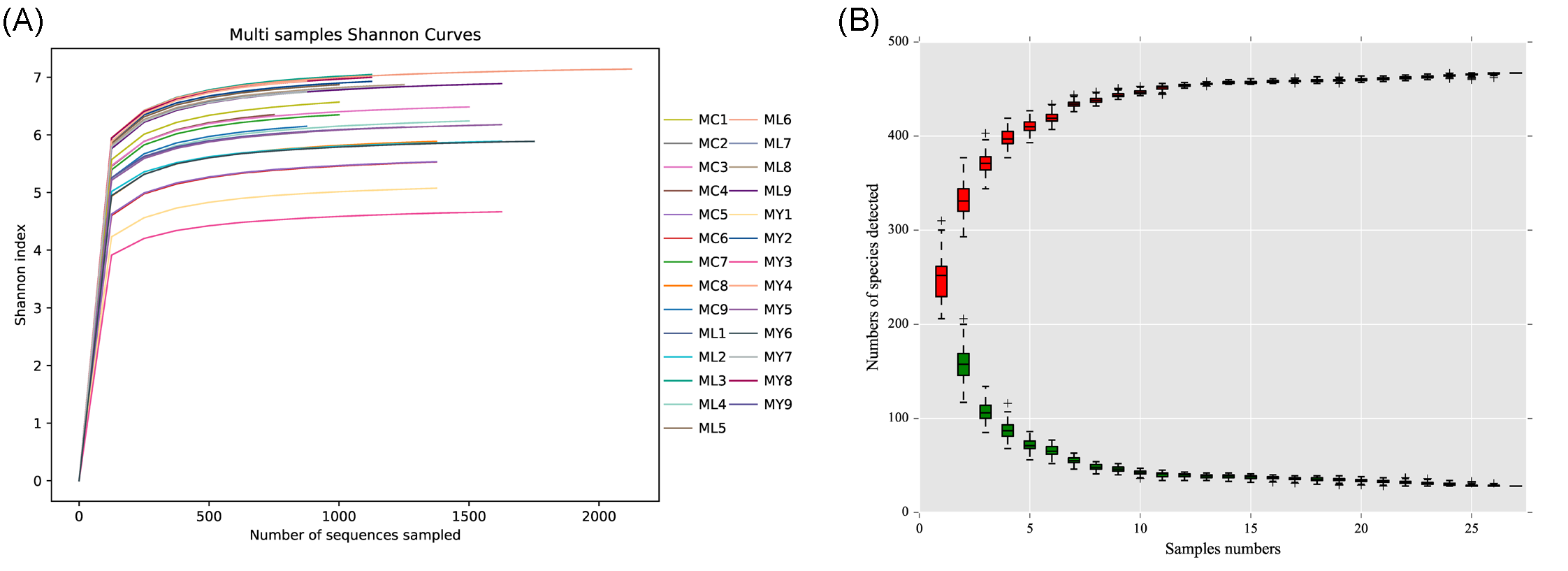


**Figure S2.** Alpha diversity curve. (A) the Shannon index rarefaction curves and (B) Species relative abundance accumulation curve (OUT level). A single red box reflects the total number of species contained in the sample, and the total red box constitutes a cumulative curve. A single green box reflects the number of common species in the sample; The total green box constitutes the common quantity curve.MC, control group (n = 9); ML, active dry yeast group (n = 9); MY, yeast culture group (n = 9).


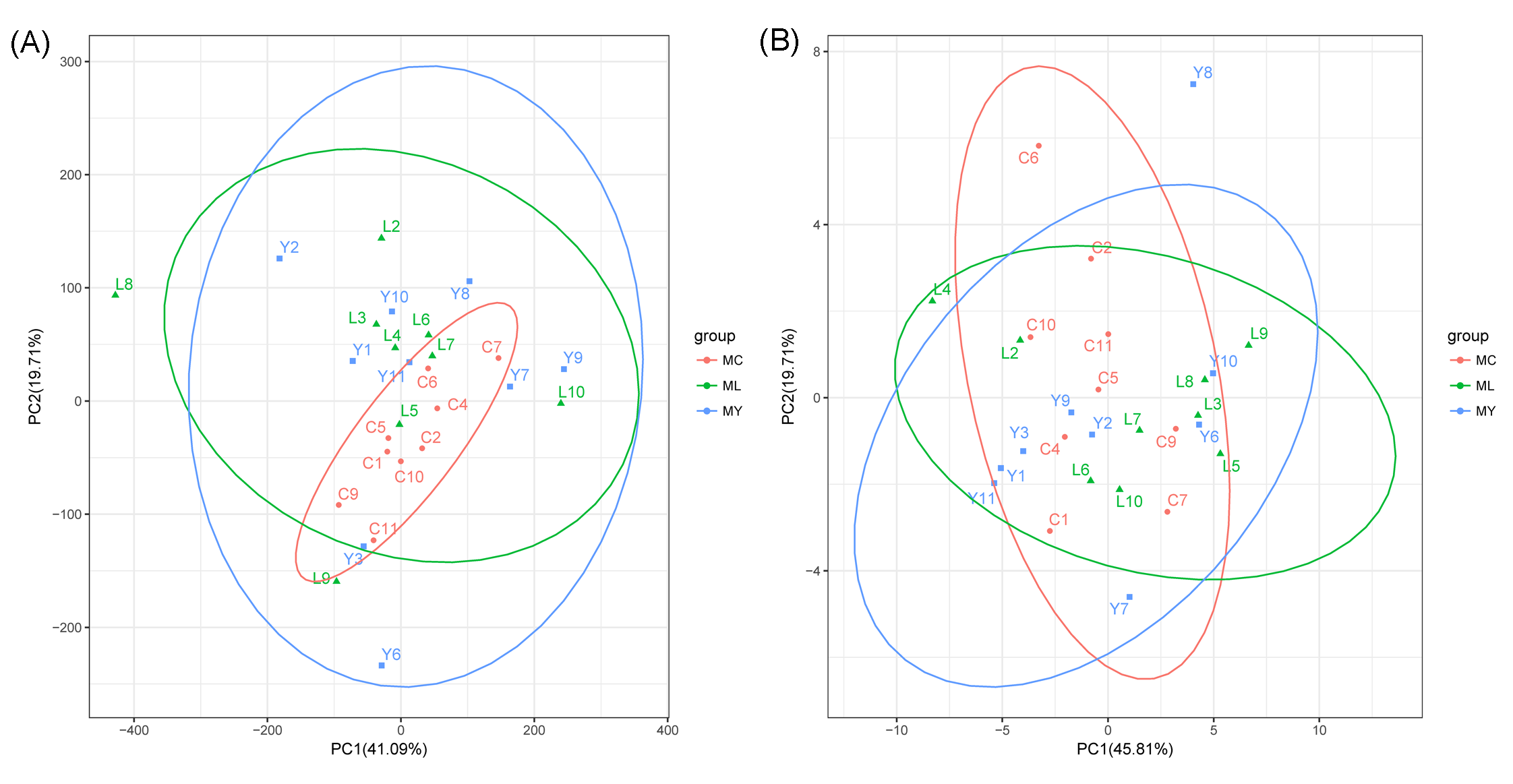


**Figure S3.** Principal component analysis (PCA) score plots of Rumen metabolites with positive (A) and negative (B) ion modes. MC, control group (n = 9); ML, active dry yeast group (n = 9); MY, yeast culture group (n = 9).


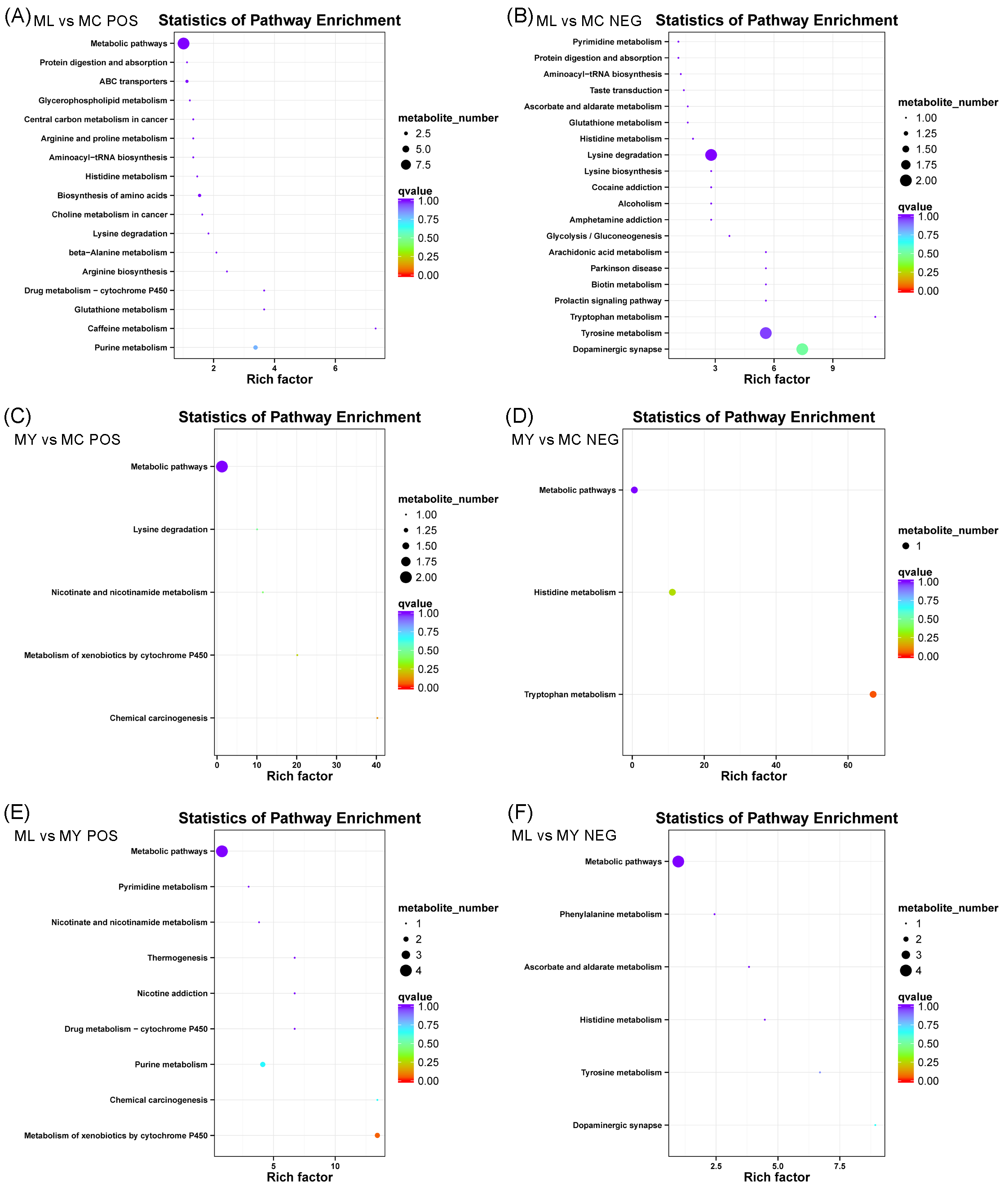


**Figure S4.** Differential metabolic pathway enrichment analysis of significantly differential metabolites in rumen. (A) and (B) for ML vs MC; (C) and (D) for MY vs MC; (E) and (F) for ML vs MY. POS, positive ion mode; NEG, negative ion mode. MC, control group (n = 9); ML, active dry yeast group (n = 9); MY, yeast culture group (n = 9).


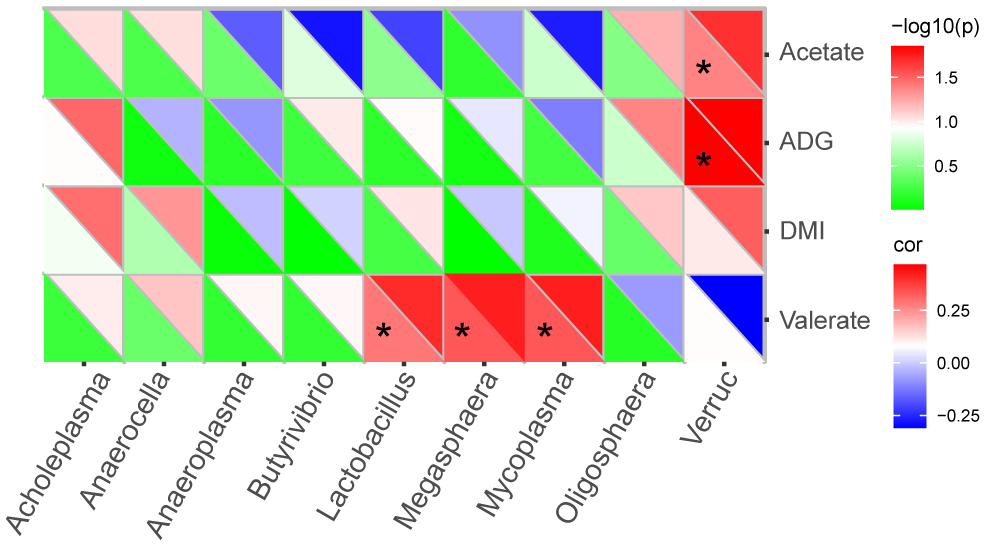


**Figure S5.** Spearman’s rank correlations between differential rumen bacteria and dietary dry matter intake (DMI), average daily gain (ADG), valerate molar percentage, and acetate molar percentage. Spearman’s rank correlation coefficient (r) was from -1 to 1. r > 0 and < 0 represented a positive and negative correlation, respectively. The (r) value denoted the degree of correlation between variables. * *P* < 0.05. MC, control group (n = 9); ML, active dry yeast group (n = 9); MY, yeast culture group (n = 9).

1. Geng, C. Y., Meng, Q. X., Ren, L. P., Zhou, Z. M., Zhang, M., & Yan, C. G. (2016). Comparison of ruminal fermentation parameters, fatty acid composition and flavour of beef in finishing bulls fed active dry yeast (Saccharomyces cerevisiae) and yeast culture. Animal Production Science, 58(5), 841-847. [↑](#footnote-ref-1)
2. Geng, C. Y., Ren, L. P., Zhou, Z. M., Chang, Y., & Meng, Q. X. (2016). Comparison of active dry yeast (Saccharomyces cerevisiae) and yeast culture for growth performance, carcass traits, meat quality and blood indexes in finishing bulls. Animal Science Journal, 87(8), 982-988. [↑](#footnote-ref-2)
